# Approaching Disordered Quantum Dot Systems by Complex Networks with Spatial and Physical-Based Constraints

**DOI:** 10.3390/nano11082056

**Published:** 2021-08-12

**Authors:** Lucas Cuadra, José Carlos Nieto-Borge

**Affiliations:** 1 Department of Signal Processing and Communications, University of Alcalá, 28801 Alcalá de Henares, Spain; 2 Department of Physics and Mathematics, University of Alcalá, 28801 Alcalá de Henares, Spain; josecarlos.nieto@uah.es

**Keywords:** quantum dot, disordered system of quantum dots, complex networks, spatial networks, quantum transport, quantum dot intermediate-band solar cells

## Abstract

This paper focuses on modeling a disordered system of quantum dots (QDs) by using complex networks with spatial and physical-based constraints. The first constraint is that, although QDs (=nodes) are randomly distributed in a metric space, they have to fulfill the condition that there is a minimum inter-dot distance that cannot be violated (to minimize electron localization). The second constraint arises from our process of weighted link formation, which is consistent with the laws of quantum physics and statistics: it not only takes into account the overlap integrals but also Boltzmann factors to include the fact that an electron can hop from one QD to another with a different energy level. Boltzmann factors and coherence naturally arise from the Lindblad master equation. The weighted adjacency matrix leads to a Laplacian matrix and a time evolution operator that allows the computation of the electron probability distribution and quantum transport efficiency. The results suggest that there is an optimal inter-dot distance that helps reduce electron localization in QD clusters and make the wave function better extended. As a potential application, we provide recommendations for improving QD intermediate-band solar cells.

## 1. Introduction

**Figure 1:**
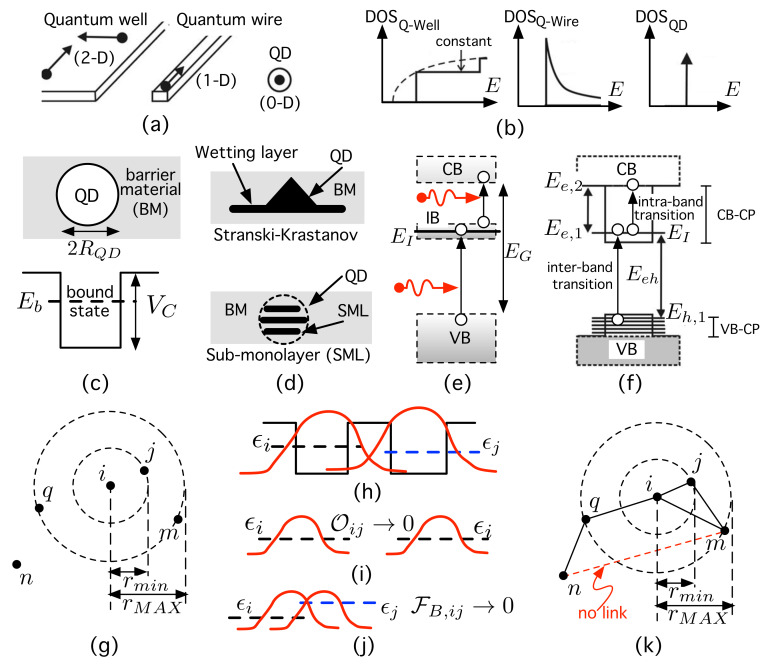
(**a**) Illustration of low-dimensional nanostructures. (**b**) Corresponding density of states (DOS). (**c**) Simplified quantum dot (QD). (**d**) Different classes of growth in self-assembled QDs. (**e**) Three electron gases in an intermediate-band solar cell. (**f**) QD and energy levels. (**g**) Distribution of QDs (=nodes). (**h**) Allowed electron hopping situation. (**i**,**j**) Forbidden electron hopping cases. (**k**) Link generation in the spacial node distribution in (**g**) according to the processes illustrated in (**h**,**i**). See the main text for further details.

Low-dimensional nanomaterials are systems that confine quantum particles in at least one of the Euclidean dimensions (D). As illustrated in Figure 1a, quantum wells, quantum wires and quantum dots (QDs) confine particles in 2-D, 1-D and 0-D, respectively. Specifically, a QD [1,2] is a “tiny” (shorter than the de Broglie wavelength) 0-D nanostructure that confines carriers in all three directions in space [1,3,4], mimicking an “artificial atom”. QDs lead to a delta-like density of states (DOS) [1], which is very different from those corresponding to the other low-dimensional structures [1] (Figure 1b). Put simply, a semiconductor QD is a heterostructure [5] formed by a small piece (∼10–15 nm in size) of a semiconductor material (“dot material” (DM)) embedded inside another with a higher bandgap (“barrier material” (BM)). This leads to the formation of confinement potentials (CPs) that confine particles, as illustrated in Figure 1c. This creates discrete or bound energy levels for carriers and modifies both the electronic and optical properties [3] when compared to those of both bulk and other nano-materials [1].

All these properties would not be useful if there were no techniques to manufacture a high density of QDs. The most successful methods are the self-assembled QD (SAQD) growth technologies [6] illustrated in Figure 1d. In the Stranski–Krastanow (SK) mode [7], the deposition of the DM starts with the formation of a very thin 2-D wetting layer, and when a critical amount of strained dot material has been deposited, the formation of (usually) pyramidal QDs occurs by relaxing strain. Conversely, sub-monolayer (SML)-QDs [8] can be formed as disks or spherical QDs [7]. SML-QDs exhibit some advantages over SK-QDs, such as a smaller diameters (5–10 nm), a higher dot density (∼$5\times 10^{11}$ cm${}^{-2}$), and better control of QD size [8,9]. SAQD technologies are crucial for implementing devices such as QD-based light-emitting diodes (LEDs) [10], QD-lasers [11,12,13,14], QD-infrared photodetectors [7,15,16], QD-solar cells [17], or QD-memories [4,18]. A key point for all these devices is that the position of carrier level(s) can be tuned by controlling the dot size [1], this being achieved by modifying the growth conditions [5,8,9,19].

Thanks to the aforementioned QD size control, QDs have made it possible to put into practice innovative photovoltaic concepts such as intermediate-band solar cells (IBSCs) [20]. These contain an intermediate band (IB) inside the semiconductor gap $E_{G}$ (Figure 1e) that allows the additional absorption of sub-bandgap photons, increasing the photocurrent without degrading the voltage. The IB (or possibly a collection of intermediate levels) is obtained by using arrays of QDs like the one in Figure 1f), leading to the quantum dot intermediate-band solar cell (QD-IBSC) [21]. This is the first device on which it has been possible to experimentally prove the concepts that the IBSC is based on [22,23,24,25]. A sufficiently dense, ordered array of QDs would lead to an IB material and would allow the electron wave function to be extended, thus favoring radiative recombination to the detriment of non-radiative recombinations. Nonetheless, although SAQD technologies allow a high density of QDs to be obtained, such QDs are neither identical nor perfectly ordered. Disorder and electron localization in QD clusters in excessively highly dense layers could degrade the efficiency. In particular, the absorption of sub-bandgap photons pumping electrons from the IB to the conduction band (CB) has been found to be too weak, possibly because it involves the gradient of a confined state at an intermediate level and an extended state in the CB. Disorder could affect not only the operation of the QD-IBSC but also that of QD-infrared photodetectors since both involve inter-sub-band or intra-band transitions (between $E_{e,1}$ and $E_{e,2}$ in Figure 1f)).

With this motivation in mind, our object of study is a special kind of disordered system of QDs. We speak of disorder in two senses: on the one hand, QDs are placed randomly with the only restriction that there is a minimum Euclidean distance ($d_{E,min}=r_{min}$) between dot centers (Figure 1g); on the other hand, QDs are not identical: during the growth process, each individual QD may have a slightly different size. This makes the electron energy level vary randomly from one dot to the other (Figure 1h). Our approach consists of considering the QD system as a complex network (CN), whose novel details are specified in subsequent paragraphs. A CN is represented by a graph [26], a set of entities called nodes (or vertices) that are connected (related in some way) to each other by means of links (or edges) [27]. In our approach, any QD is represented by a node (Figure 1g), while the possible hopping of an electron from QD *i* (with energy level $\epsilon_{i}$) to another one *j* (with energy level $\epsilon_{j}$), as shown in Figure 1h, is encoded by using a weighted link:

(1) $$w_{ij}=O_{ij}\cdot F_{B,ij},$$

where

(2) $$O_{ij}=\int_{V}\psi_{QD_{j}}^{*}\psi_{QD_{i}}\;dr$$

is the overlap integral [28] (computed over all volume *V*) between the electron wave function $\psi_{QD_{i}}$ and $\psi_{QD_{j}}$ at nodes *i* and *j*, respectively, and

(3) $$F_{B,ij}=\exp\left(\frac{\epsilon_{i}-\epsilon_{j}}{k_{B}T}\right)$$

is a Boltzmann factor at temperature *T*, with $k_{B}$ being the Boltzmann constant. Intuitively, far QDs with null overlap ($O_{ij}=0$ in Figure 1i) or close QDs but with large energy difference ($F_{B,ij}=0$ in Figure 1j) lead to no electron hopping: the weight link is $w_{ij}=0$. This is the case for QDs *m* and *n* in Figure 1j, between which there is no link.

Many approaches have been proposed to study systems with extremely different natures as almost all systems can be modeled as sets of entities that interact with each other [27]. Because of this versatility, CN Science has become a multidisciplinary approach to study structural relationships in many systems [29,30], ranging from human-made systems (power grids [31,32], the Internet [33], and transportation networks [34], etc.) to natural systems (ecosystem [35], vascular networks [36], metabolic networks [37], and others [27]). The extensive set of CN mathematical tools [38,39] helps us to understand epidemic processes [40] such as COVID-19 [41], information spreading [42,43], or cascading failures in technological networks [44,45]. Although CN Science has been applied to a vast variety of macroscopic systems, it has been used to a much lesser extent to study quantum nanosystems and, when this has been performed, it has been used in a conceptually different way from the approach we propose in this paper, as shown in Section 2.

The novelty of our proposal, especially in relation to that in [46], is threefold. First, although we consider that the QDs are randomly distributed in a metric space (spatial network (SN) [34,47]), they have to fulfill the condition that there is a minimum inter-dot distance that cannot be violated (to avoid localization effects, as described below). Second, our model allows nodes with different attributes—in particular, different energy levels—to be considered. Third, the link formation and weighting process that we propose are consistent with the laws of quantum physics and statistics: it not only takes into account the overlap integral but also Boltzmann factors to include the fact that an electron can hop from one QD to another with a slightly different energy level.

The rest of this paper has been structured as follows. After reviewing of the current state of the art in Section 2, Section 3 provides a theoretical background that, based on the quantum Lindblad master equation, naturally leads to the use of Boltzmann factors. Section 4 briefly introduces the QD system that we want to study and how the corresponding network could be generated. The experimental work in Section 5 allows us to predict inner features of the system such as the system quantum state, its time evolution, or to what extent quantum transport is important. The main results suggest that there is an optimal inter-dot distance that helps to reduce electron locations in QD clusters and make the wave function better extended. Section 6 discusses a potential application in QD-IBSCs with the aim of increasing the weak absorption of photons causing electron transitions from intermediate states in QDs to the conduction band. Finally, Section 7 completes the paper with a summary and the main conclusions.

## 2. Related Work

There are some works that apply CN concepts to explore nanosystems governed by the laws of Quantum Mechanics (QM) [48] and not by those of classical physics. A representative example is the system studied in [49]: any atom trapped in a cavity is represented by a node, while the photon that the two atoms (nodes) exchange is encoded by a link between them. This and other papers have in common the fact of studying quantum properties on networks using the concept of quantum walk (QW). This is because the quantum dynamics of a discrete system can be re-expressed and interpreted as a single-particle quantum walk [50,51]. This is the reason why quantum walks have been used to study the transport of energy through biological complexes involved in light harvesting in photosynthesis [52]. Quantum walks have also been used to explore transport in systems described by CN with different topologies [53,54]. Specifically, continuous-time quantum walks (CTQW)—a class of quantum walks in continuous time and discrete space [55]—have been used extensively to study quantum transport (QT) on CN [54] and are also used in our work. There are several works that have studied QT over regular lattices [54,56,57], branched structures [58,59] (including dendrimers [59]), fractal patterns [60], Husimi cacti [61], Cayley trees [62], Apollonian networks [63], scale-free networks [64], small-world (SW) networks [65], and start graphs [66,67], leading to the conclusion that QT differs from its classical counterpart. Having a quantitative measure of the efficiency of QT in a CN has been found to be important for practical and comparative purposes. In this regard, in [68], bounds were found that allow for the measurement of the global transport efficiency of CN, defined by the time-averaged return probability of the quantum walker. QT efficiency can undergo abrupt changes and can have transitions from localization (no QT) to delocalization (QT appears). In this respect, the authors of [69] have studied the localization–delocalization transition of electron states in SW networks. The SW feature is interesting because it makes it easy to navigate a network as SW networks exhibit a relatively short path between any pair of nodes [70,71]: the mean topological distance or average path length *ℓ* is small when compared to the total number of nodes *N* in the network (ℓ=O(lnN) as N→∞). The usual techniques of rewiring [70] or adding links [72] in macroscopic, non-quantum CN to create SW networks have also been extended to quantum systems [65,67] to enhance QT. In [65], SW networks have been generated from a one-dimensional ring of *N* nodes by randomly introducing *B* additional links between them. The dynamics of quantum particles have been modeled by CTQW, computing the averaged transition probability to reach any node of the network from the initially excited node. Finally, the strategy of adding new links has been explored in star networks with the aim of enhancing the efficiency of quantum walks to navigate the network [67]. Please note that all these key works have focused their research on the perspective of the topological properties; in particular, the topological (geodesic) distance between two nodes *i* and *j*, d(i,j), is the length of the shortest path (geodesic path) between them—that is, the minimum number of links when moving from one node to the other [73]. The distance between two nodes *i* and *j* that are directly linked is d(i,j)= 1, regardless of where they are located in physical space.

A conceptually different approach has recently been proposed in [46], as it focuses on modeling a disordered ensemble of QDs as Random Geometric Graphs (RGG) with weighted links, with these being the overlap integral (or electron probability amplitude) between the QDs (=nodes) involved. These are networks in which the nodes are spatially embedded [74] or constrained to sites in a metric space, usually, the Euclidean distance $d_{E}$. This particular subset of networks is called the spatial network (SN) [34,47] or spatially embedded CN [75]. A particular class of SNs are Random Geometric Graphs (RGGs) [76], which have successfully been used to model wireless sensor networks [77] and ad hoc networks [78].

## 3. Theoretical Framework

As mentioned in Section 1, the quantum system studied here is made up of many quantum dots embedded in a (barrier) semiconductor. The problem at hand can be considered in the framework of open quantum systems [79]. The quantum dot subsystem, called *S* from now on, is an open system that exchanges energy with its environment. The total or global system is assumed to be closed or isolated. Usually, the dynamics of an open quantum system are described in terms of the reduced density operator, which is obtained from the density operator of the total system by tracing over the variables of the environment [79]. With the aim of tackling this problem, the use of a number of approximations leads to an equation of motion for the density matrix of the open system, as shown in Section 3.2. Before that, it is necessary to delve into the key concept of density operator, which is briefly introduced in Section 3.1 using the notation presented by Cohen–Tannoudji in [28].

### 3.1. The Density Operator

There are quantum systems about which we have incomplete information. In quantum mechanics, such incomplete information usually appears as follows: the state of the system may be either the state $|\psi_{1}\rangle$ with a probability $p_{1}$, or the state $|\psi_{2}\rangle$ with a probability $p_{2}$, ⋯, and so on. These probabilities fulfill $p_{1}+p_{2}+\cdots =\sum_{k}p_{k}=1$. In this general case, it is said that we are dealing with a *statistical mixture* of states $|\psi_{1}\rangle$, $|\psi_{2}\rangle$, ⋯, with probabilities $p_{1}$, $p_{2}$, ⋯. Note that the simplest case is that in which the state of the system is perfectly known (all the probabilities $p_{k}$ are zero, except one). The system is then said to be in a *pure state* [28].

Let us assume that the system state is a statistical mixture. The density operator is defined as [28]

(4) $$\hat{\rho }=\sum_{k}p_{k}|\psi_{k}\rangle \langle \psi_{k}|,$$

with its dynamics ruled by the von Neumann equation (also known as the Liouville–von Neumann equation),

(5) $$\frac{d}{dt}\hat{\rho }=\frac{i}{\hbar }\left[\hat{\rho },\hat{H}\right].$$

To fully understand the physical meaning of the density operator $\hat{\rho }$, it is convenient to examine its matrix elements. Let $\left\{|u_{n}\rangle \right\}$ be an orthonormal basis of the vector space. The diagonal elements of the density matrix are

(6) $$\rho_{nn}=\langle u_{n}|\rho |u_{n}\rangle ,$$

meaning that, by substituting Equation (4) in (6), we obtain that

(7) $$\rho_{nn}=\sum_{k}p_{k}\langle u_{n}|\psi_{k}\rangle {\langle u_{n}|\psi_{k}\rangle }^{*}=\sum_{k}p_{k}{\left|\langle u_{n}|\psi_{k}\rangle \right|}^{2}.$$

This means that if the state of the system is $|\psi_{k}\rangle$, then ${\left|\langle u_{n}|\psi_{k}\rangle \right|}^{2}$ is the probability of finding (in a measurement) the system in the state $|u_{n}\rangle$. $\rho_{nn}$ represents the average probability of finding the system in the state $|u_{n}\rangle$. This is the reason why $\rho_{nn}$ is called the *population* of the state $|u_{n}\rangle$: if the same measurement is carried out *N* times under the same initial condition, if *N* is a large number, then a number of $N\rho_{nn}$ systems will be found in the state $|u_{n}\rangle$ (see [28] for details).

On the other hand, the off-diagonal elements, again using Definition (4), are

(8) $$\rho_{nm}=\langle u_{n}|\rho |u_{m}\rangle =\sum_{k}p_{k}\langle u_{n}|\psi_{k}\rangle \langle \psi_{k}|u_{m}\rangle ,$$

where the cross-term $\langle u_{n}|\psi_{k}\rangle \langle \psi_{k}|u_{m}\rangle$ represents interference effects between the state $|u_{n}\rangle$ and $|u_{m}\rangle$, which can occur when the state $\psi_{k}$ is a coherent linear superposition of these states.

Note in (8) that $\rho_{nm}$ is a sum of complex numbers. If $\rho_{nm}=0$, this means that the average in (8) has canceled out any interference effect between $|u_{n}\rangle$ and $|u_{m}\rangle$. On the contrary, if $\rho_{nm}\neq 0$, a given coherence subsists between these states. This is the reason why the non-diagonal elements of the density operator in $\hat{\rho }$ are usually called *coherences*.

The distinction between population and coherences depends on the basis chosen $\left\{|u_{n}\rangle \right\}$ in the state space. Since $\hat{\rho }$ is Hermitian, then it is *always* possible [28] to find an orthonormal basis $\left\{|\chi_{q}\rangle \right\}$ where $\hat{\rho }$ is diagonal. As a consequence, $\hat{\rho }$ describes a statistical mixture of the states $|\chi_{q}\rangle$ with the probabilities $\pi_{q}$,

(9) $$\hat{\rho }=\sum_{q}\pi_{q}\;|\chi_{q}\rangle \langle \chi_{q}|.$$

Since $\hat{\rho }$ is positive and tr$\left(\hat{\rho }\right)$=1, then $0\leq \pi_{k}\leq 1$ and $\sum_{k}\pi_{k}=1$.

In the particular case in which the kets belonging to the orthogonal base $\left\{|u_{n}\rangle \right\}$ are the eigenvectors $\left\{|\phi_{n}\rangle \right\}$ of the Hamiltonian *H*,

(10) $$\hat{H}|\phi_{n}\rangle =E_{n}|\phi_{n}\rangle ,$$

the corresponding matrix elements for the von Neumann Equation (5) are [28]

(11) $$\left\{\begin{array}{cc} \frac{d}{dt}\rho_{nn}\left(t\right)=0 & \\ \frac{d}{dt}\rho_{np}\left(t\right)=\frac{i}{\hbar }\left(E_{n}-E_{p}\right)\;\rho_{nn}\left(0\right) & \end{array}\right.$$

This leads to a solution for the von Neumann equation as follows:

(12) $$\left\{\begin{array}{cc} \rho_{nn}\left(t\right)=\mathrm{constant} & \\ \rho_{np}\left(t\right)=e^{\frac{i}{\hbar }\left(E_{p}-E_{n}\right)t}\rho_{np}\left(0\right) \end{array}\right.$$

This solution for the von Neumann equation shows that the populations are constant, while the coherences oscillate at the Bohr frequencies of the quantum system [28].

### 3.2. Electron Dynamics of the Open Quantum System S

As introduced in Section 1, let us now consider our quantum mechanical system formed by a layer of *N* QDs embedded inside a barrier bulk semiconductor. This quantum mechanical system *S* is an open system [79] that is in thermodynamic equilibrium with an environment (*E*), reservoir, or heat bath at a given temperature *T*. The environment *E* models the bulk barrier material into which the QD layer is embedded. Sometimes, the environment of the open system is called a reservoir to denote an environment with an infinite number of degrees of freedom. If the reservoir is in thermal equilibrium, one usually speaks of a heat bath [79].

Furthermore, an open quantum system is a quantum mechanical system *S* with Hilbert space $H_{S}$ which is coupled to another system *E*, the environment, with Hilbert space $H_{E}$. Thus, we can view *S* as a subsystem of the total system S+E residing in the space $H_{T}=H_{S}⊗H_{E}$. A relevant and useful feature of an open system is the fact that all observables $\hat{A}$ of interest refer to this system. Any of these observables can be written in the form $\hat{A}⊗{𝟙}_{E}$, where $\hat{A}$ acts in $H_{S}$, the Hilbert space of the open system *S* [79]. If the state of the total system S+E is described by some density operator $\hat{\rho }$, then the expectation value of the observable $\hat{A}$ is

(13) $$\langle \hat{A}\rangle ={\mathrm{tr}}_{S}\left\{\hat{A}\;{\hat{\rho }}_{S}\right\},$$

where

(14) $${\hat{\rho }}_{S}={\mathrm{tr}}_{E}\;{\hat{\rho }}_{T}$$

is the reduced density matrix operator and ${\hat{\rho }}_{T}\left(t\right)$ is the density operator of the total system. In Equations (13) and (14), tr${}_{S}$ and tr${}_{E}$ stand for, respectively, the partial traces over the degrees of freedom of the open system *S* and of the environment *E*. The reduced density matrix is of key practical importance for exploring open quantum systems [79].

Depending on the strength of the coupling between the system and the environment, the dynamics in *S* could change from quantal to classical [54]. Regarding this, the Hamiltonian of the total system ${\hat{H}}_{T}$ is

(15) $${\hat{H}}_{T}={\hat{H}}_{S}+{\hat{H}}_{E}+{\hat{H}}_{S-E},$$

where ${\hat{H}}_{S}$ and ${\hat{H}}_{E}$ are the Hamiltonians of *S* and *E*, respectively, and ${\hat{H}}_{S-E}$ is the Hamiltonian for the coupling between the system and the environment. Note that the total system belongs to a Hilbert space $H_{T}=H_{S}⊗H_{E}$ with a huge dimension (because of the many degrees of freedom of the environment) [54]. This problem can be overcome thanks to quantum mechanical formulations [80,81,82] that prove that there exists an equilibrium Hilbert subspace $H_{eq}$ for which any initial state approaches equilibrium within a very short time. In particular, in [82], the electron reaches its stationary state extremely quickly, with a time in the order of the Boltzmann time, $\tau_{B}∼1.6\times 10^{-13}$ s at T∼300 K.

As we are interested in the electron dynamics in the QD system, we have adopted an approach that consists of tracing over the environmental degrees of freedom to obtain the reduced density matrix operator of the system [80,81,82,83], as stated in Equation (14).

The dynamics of ${\hat{\rho }}_{S}\left(t\right)$ can be studied by using the quantum Lindblad master equation (LME) [83] because it preserves the density matrix positivity [84]. Under weak coupling between the system and the environment, the LME can be expressed as

(16) $$\frac{d{\hat{\rho }}_{S}\left(t\right)}{dt}=\underset{\mathrm{unitary}\;\mathrm{part}}{\underbrace{\frac{i}{\hbar }\left[{\hat{\rho }}_{S}\left(t\right),{\hat{H}}_{S}\right]}}+\underset{\mathrm{incoherent}\;\mathrm{part}}{\underbrace{\sum_{k}\gamma_{k}\left({\hat{A}}_{k}\hat{\rho }\left(t\right){\hat{A}}_{k}^{†}-\frac{1}{2}\left\{{\hat{A}}_{k}^{†}{\hat{A}}_{k},\hat{\rho }\left(t\right)\right\}\right)}},$$

where the anticonmutator $\left\{\cdot \right\}$ is defined as $\left\{a,b\right\}≐ab+ba$, while ${\hat{A}}_{k}$ and ${\hat{A}}_{k}^{†}$ are the jump or transition operators. They account for the transitions between state pairs in *S* (induced by system–bath interactions) with rates $\gamma_{k}$. The incoherent part (IP) of the LME can be rewritten [83] using the notation stated in [84] as

(17) $$\mathrm{IP}\equiv \sum_{k>j}\gamma_{k\to j}\left({\hat{A}}_{jk}\hat{\rho }\left(t\right){\hat{A}}_{jk}^{†}-\frac{1}{2}\left\{{\hat{A}}_{jk}^{†}{\hat{A}}_{jk},\hat{\rho }\left(t\right)\right\}\right)+\sum_{k\leq j}\gamma_{k\to j}\left({\hat{A}}_{kj}\hat{\rho }\left(t\right){\hat{A}}_{kj}^{†}-\frac{1}{2}\left\{{\hat{A}}_{kj}^{†}{\hat{A}}_{kj},\hat{\rho }\left(t\right)\right\}\right)$$

where each transition operator is defined as [84]

(18) $${\hat{A}}_{jk}=|\phi_{j}\rangle \langle \phi_{k}|.$$

Note that the ket $|\phi_{n}\rangle$ is the eigenstate of the Hamiltonian ${\hat{H}}_{S}$

(19) $${\hat{H}}_{S}|\phi_{n}\rangle =\epsilon_{n}|\phi_{n}\rangle ,$$

with $\epsilon_{n}$ being the energy corresponding to state $|\phi_{n}\rangle$. $\left\{|\phi_{n}\rangle \right\}$ is the corresponding orthonormal basis of $H_{S}$.

To advance further in computing the matrix elements, we apply the operation $\langle \phi_{n}|•|\phi_{n}\rangle$ on Equation (16) (where • represents the operator), and after this, we apply $\langle \phi_{n}|•|\phi_{m}\rangle$ on Equation (16).

On the one hand, when applying $\langle \phi_{n}|•|\phi_{n}\rangle$ on Equation (16), we reach

(20) $$\frac{d\langle \phi_{n}|\hat{\rho }\left(t\right)|\phi_{n}\rangle }{dt}\equiv \frac{d}{dt}\rho_{nn}\left(t\right)=\sum_{j}\left(\gamma_{n\to j}p_{j}\left(t\right)-\gamma_{j\to n}p_{n}\left(t\right)\right).$$

This expression is similar to that obtained in [84]. Note in Equation (20) that $p_{i}$ is the electron probability at state $|\phi_{i}\rangle$ under the Boltzmann distribution

(21) $$p_{i}=\frac{1}{Q}\exp\left(-\frac{\epsilon_{i}}{k_{B}T}\right),$$

where *Q* is the canonical partition function. Note that the canonical ensemble represents the possible states of system *S* in thermal equilibrium with the heat bath *E* at a fixed temperature *T* (*S* exchanges energy with the heat bath *E*).

The stationary state in Equation (20) is reached when $\gamma_{n\to j}p_{j}\left(t\right)-\gamma_{j\to n}p_{n}\left(t\right)=0$; that is, when $\gamma_{n\to j}p_{j}\left(t\right)=\gamma_{j\to n}p_{n}\left(t\right)$. This means that the transition rates should be related by the detailed balance condition

(22) $$\frac{\gamma_{n\to j}}{\gamma_{j\to n}}=\exp\left(\frac{\epsilon_{j}-\epsilon_{n}}{k_{B}T}\right)\equiv F_{B,ij},$$

which also makes the incoherent part of Equation (16) zero, and thus Equation (20) becomes

(23) $$\frac{d}{dt}\rho_{nn}\left(t\right)=0.$$

On the other hand, when we now apply $\langle \phi_{n}|•|\phi_{m}\rangle$ on Equation (16), we obtain

(24) $$\frac{d\langle \phi_{n}|\hat{\rho }\left(t\right)|\phi_{m}\rangle }{dt}\equiv \frac{d}{dt}\rho_{nm}\left(t\right)=\frac{i}{\hbar }\left(E_{n}-E_{p}\right)\;\rho_{nn}\left(0\right)+0.$$

The zero value that we have explicitly written on the right side of Equation (24) corresponds to the incoherent part and arises from the fact that, when applying $\langle \phi_{n}|•|\phi_{m}\rangle$ on Equation (17), $\langle \phi_{n}|$ IP $|\phi_{m}\rangle$ always has (in all its addends) sums of the type

(25) $$\sum_{k,j}\gamma_{kj}\langle \phi_{n}|\phi_{j}\rangle \langle \phi_{k}|\phi_{k}\rangle \langle \phi_{j}|\phi_{m}\rangle =0.$$

This is because, when j=n then $\langle \phi_{n}|\phi_{n}\rangle =1$ but $\langle \phi_{n}|\phi_{m}\rangle =0$, and when j=m, then $\langle \phi_{n}|\phi_{m}\rangle =0$ and $\langle \phi_{m}|\phi_{m}\rangle =1$.

Finally, considering the results (23) and (24), we reach the stationary state ruled by

(26) $$\left\{\begin{array}{cc} \frac{d}{dt}\rho_{nn}\left(t\right)=0 & \\ \frac{d}{dt}\rho_{np}\left(t\right)=\frac{i}{\hbar }\left(E_{n}-E_{p}\right)\;\rho_{nn}\left(0\right) & \end{array}\right.$$

which leads to the solution

(27) $$\left\{\begin{array}{cc} \rho_{nn}\left(t\right)=\mathrm{constant} & \\ \rho_{np}\left(t\right)=e^{\frac{i}{\hbar }\left(E_{p}-E_{n}\right)t}\rho_{np}\left(0\right) \end{array}\right.$$

This is the same solution given by Equation (12), corresponding to the solution of the von Neumann equation in an isolated quantum system.

Please note that the fact that the incoherent part of the LME (16) vanishes has been obtained under a set of assumptions: (1) weak coupling between the system and the environment [83,84]; (2) at t=0, the system and the environment are uncorrelated and have a separable state in the form $\rho_{T}\left(0\right)=\rho_{S}\left(0\right)⊗\rho_{E}\left(0\right)$ [83]; (3) the initial state of the environment is thermal, meaning that it is described by a density matrix in the form $\rho_{E}\left(0\right)=\exp\left(-H_{E}/\left(k_{B}T\right)\right)/\mathrm{tr}\left(\exp\left(-H_{E}/\left(k_{B}T\right)\right)\right)$ [83]; and (4) $\gamma_{n\to j}p_{j}\left(t\right)-\gamma_{j\to n}p_{n}\left(t\right)=0$ [84]. Under this strict set of assumptions, the incoherent part of the system approaches zero, which suggests that the electron dynamics are coherent.

The detailed balance argument leading to Equation (22) is the reason why we have considered Boltzmann factors $F_{B,nj}$ in Equation (3) to form links. The electron system *S*, the QD system under study, is an open system in thermodynamic equilibrium with a much bigger bath *E* at temperature *T*: when the electron makes a transition from a QD with energy $\epsilon_{n}$ to another with energy $\epsilon_{j}>\epsilon_{n}$, the energy difference is $\Delta E=\epsilon_{n}-\epsilon_{j}$, which is supplied by the environment. The opposite is also true using the detailed balance concepts presented in Equation (22).

## 4. Approaching the QD System by a Network with Spatial and Physical-Based Constraints

Before describing the system *S*, which consists of many QDs, it is convenient to consider some important properties of a single, isolated QD (Section 4.1), which will assist us in better describing the system *S* in Section 4.2.

### 4.1. A Single QD

We begin our reasoning by considering a single quantum dot. We compute the electron wave function of a bound state and its corresponding energy. This wave function will be useful to understand how different dots interact with each other. To do this, we consider a set of simplifying hypotheses.

We first assume that the single-band effective mass equation of electrons in the envelope approximation [85] is a proper description of the dot and barrier materials. This is because a QD size of 10 to 20 nm is much larger than the lattice constant of the material involved, and thus it seems reasonable to consider that only the envelope part of the electron wave function is affected by the confinement potential. This is the so-called envelope function approximation. Its name arises from the conclusion that the physical properties can be derived from the slowly varying envelope function ψ rather than the total wave function [1],

(28) $$\Psi \approx \psi \left(r\right)\cdot u_{C}\left(r\right)$$

where $u_{C}\left(r\right)$, the periodic part of the Bloch function in the CB, is rapidly varying on the scale of the crystal lattice. $u_{C}\left(r\right)$ is also assumed to be independent of the wave-vector (k) of the reciprocal crystal lattice.

We also assume spherical QDs of radius $R_{QD}$. The center of any QD *i* is given by a position vector $r_{i}$ in the metric space. We consider that its associated confinement potential is spherically symmetric (depending only on the radial co-ordinate *r*), finite, and square [86] (Figure 1c):

(29) $$U_{QD}=\left\{\begin{array}{cc} -V_{C} & ,\mathrm{if}\;r<R_{QD}\\ 0 & ,\mathrm{if}\;r>R_{QD}, \end{array}\right.$$

where the subscript “QD” means that we have only one isolated QD.

The time-independent Schrödinger’s equation (${\hat{H}}_{QD}\psi =E_{QD}\cdot \psi$) for an electron of effective mass $m_{e}^{*}$ in the the central energy potential $U_{QD}$ is

(30) $${\hat{H}}_{QD}\;\psi ≐\left(-\frac{\hbar }{2m_{e}^{*}}+U_{QD}\right)\psi =E_{QD}\cdot \psi .$$

This equation is separable [86,87] and the envelope functions for bound states in a QD characterized by the spherical three-dimensional energy potential $U_{QD}$ are described by

(31) $$\psi_{nlm}\left(r,\theta ,\varphi \right)=R_{nl}\left(r\right)Y\left(r,\theta ,\varphi \right),$$

where Y(r,θ,φ) are the spherical harmonics and $R_{nl}\left(r\right)$ is the radial function [87]. This, for l=0—to illustrate the objective of our work, it is enough to consider only one electron bound level; i.e., the ground state (GS)—allows us to write Equation (30) in the form

(32) $$-\frac{\hbar }{2m_{e}^{*}}\left(\frac{1}{r^{2}}\frac{d}{dr}\left(r^{2}\frac{d}{dr}\right)+U_{QD}\right)R\left(r\right)=E_{QD}\cdot R\left(r\right),$$

where

(33) $$m_{e}^{*}=\left\{\begin{array}{cc} m_{D}^{*} & ,\mathrm{if}\;r<R_{QD}\\ m_{B}^{*} & ,\mathrm{if}\;r>R_{QD}, \end{array}\right.$$

is the electron effective mass within the dot (D) and the barrier (B) materials, respectively. This again simplifies the problem as it avoids the formulation of continuity boundary conditions at the dot–barrier interface.

The aforementioned simplifying hypotheses allow the time-independent Schrödinger’s Equation (30) to be solved analytically [86,87,88]. In addition, the continuity of the logarithmic derivative of the electron envelope function, ψ, at the dot–barrier interface has been considered together with the boundary condition ψ→0 as r→∞ [87]. The envelope function corresponding to this GS (n=0 and l=0) is

(34) $$\psi_{QD}\equiv \psi \left(r\right)=\left\{\begin{array}{cc} Aj_{0}\left(\alpha r\right) & ,\mathrm{if}\;r<R_{QD}\\ Bh_{0}^{\left(1\right)}\left(i\beta r\right) & ,\mathrm{if}\;r>R_{QD}, \end{array}\right.$$

where $j_{0}\left(r\right)$ is the Bessel’s spherical function of zero order, $h_{0}^{\left(1\right)}\left(r\right)$ is the Hankel’s spherical function of zero order, and α and β are [87,88], respectively,

(35) $$\alpha ={\left(\frac{2m_{D}^{*}\left(E-U_{0}\right)}{\hbar }\right)}^{1/2}$$

(36) $$\beta ={\left(\frac{2m_{B}^{*}E}{\hbar }\right)}^{1/2}$$

The number of bound states in a QD depends on $V_{C}\cdot {\left(2R_{QD}\right)}^{2}$ (see [86,87]): there is a range of values of $V_{C}\cdot {\left(2R_{QD}\right)}^{2}$ for which there is only one energy level. Solving the equation

(37) $$\alpha \left(E\right)\cdot \cot\left(\alpha \left(E\right)\cdot R_{QD}\right)=-\beta \left(E\right),$$

restricted to the condition [86]

(38) $$\frac{\pi }{2}<{\left(\frac{2m_{D}^{*}}{\hbar^{2}}V_{C}R_{QD}^{2}\right)}^{\left(1/2\right)}<\frac{3\pi }{2},$$

leads to one odd bound state, whose energy *E* depends on $R_{QD}$.

**Figure 2:**
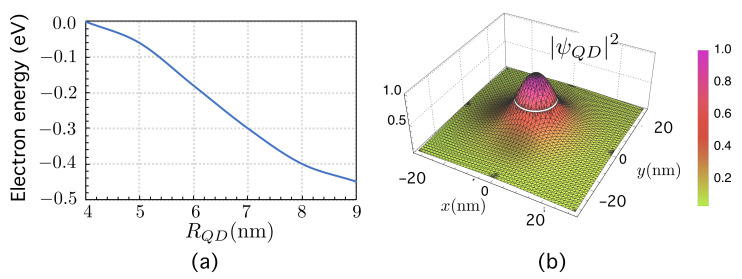
(**a**) Position of the electron energy level (eV) below the CB ($E_{C}=0$ is assumed to be as the energy reference origin) as a function of the quantum dot radius $R_{QD}$. (**b**) Square modulus of the electron wave function for $R_{QD}=8$ nm.

We have solved the problem for a single, isolated QD with $-V_{C}=0.68$ eV using electron effective masses typical in the InAs/AlGaAs material system for different values of the QD radius, $R_{QD}$, as shown in Figure 2a, together with the corresponding square modulus of the electron wave function (Figure 2b) for $R_{QD}=8$ nm.

In the simulations that follow, we have considered $R_{QD}=8$ nm, leading to an energy level E=−0.4 eV $\equiv E_{QD}\equiv E_{I}$. Its associated wave-function is a 1s−orbital [5,88]. Figure 2b shows its corresponding square module, $|\psi_{QD}{|}^{2}$, on the axes *x* and *y*.

### 4.2. The Quantum System S

Let us consider the quantum mechanical system *S* formed by a set of *N* QDs that are randomly distributed and srestricted to the condition that there is a minimum Euclidean inter-dot distance ($d_{E,min}=r_{min}$) between QD centers (Figure 1g).

We generated the dot centers as follows: first, *N* dot centers were randomly generated with a uniform distribution on a square with side *L* and finite area $A=L^{2}$. Second, the minimum distance between any two centers in the finite square with area *A* was computed. Let $d^{\left(A\right)}$ be such a distance. Third, a uniform (isotropic) scaling was carried out. The scale factor was $\alpha =r_{min}/d^{\left(A\right)}$ so that if $\left(x_{i}^{\left(A\right)},y_{i}^{\left(A\right)}\right)$ were the coordinate of a center *i* in the square with area *A*, then $\left(\alpha \;x_{i}^{\left(A\right)},\alpha \;y_{i}^{\left(A\right)}\right)$$\equiv r_{i}$ would be the center of the corresponding QD *i* in a square with area ${\left(\alpha L\right)}^{2}$. This square was a finite area located in the metric space $R^{2}$.

Let us now assume that the Hamiltonian of the system formed by *N* QDs can be approximated by a special case of a tight-binding (TB) Hamiltonian based on the very weak superposition of wave functions for isolated QDs, as in the Hückel model [89]

(39) $${\hat{H}}_{S}={\hat{H}}_{\mathrm{QD}}+\sum_{r_{n}\neq 0}^{N}V_{{\mathrm{QD}}_{n}}\left(r-r_{n}\right)={\hat{H}}_{\mathrm{QD}}+\Delta U\left(r\right),$$

where ΔU(r) is a very small perturbation over the Hamiltonian of the isolated QD located at r=0.

Using this approach, the solution ψ to the time-independent single electron Schrödinger’s equation is approximated by a linear combination of wave functions $\psi_{QD}\left(r-r_{n}\right)$, with n=1,⋯,N, in a similar approach to that of the Linear Combination of Atomic Orbitals (LCAO). Note that $\psi_{QD}\left(r-r_{n}\right)$ is simply the wave function of the single electron stated by Equation (34), but located at $r=r_{n}$.

From now on, we adopt Dirac’s notation, meaning that $\psi_{QD}\left(r-r_{i}\right)\equiv |i\rangle$. Thus, the electron state is a linear combination of the state vectors or kets,

(40) $$|\psi \rangle =\sum_{i=1}^{N}c_{i}|i\rangle ,$$

where the coefficients $c_{i}$ are computed by normalizing to unity:

(41) $$\int_{V}d^{3}r\;\psi^{*}\psi \equiv \langle \psi |\psi \rangle =1,$$

where *V* refers to the entire volume. Note that in the TB approach 〈i|i〉=1, while

(42) $$\langle i|j\rangle \equiv O_{ij}=\int_{V}d^{3}r\;\psi_{QD}^{*}\left(r-r_{i}\right)\;\psi_{QD}\left(r-r_{j}\right)\ll \langle i|i\rangle .$$

This weak-overlap hypothesis was numerically checked to validate our model and, as shown in Section 5.2, its order of magnitude was less than $10^{-2}$.

With this in mind, the TB Hamiltonian can be formally written as

(43) $${\hat{H}}_{S}=\sum_{i}\epsilon_{i}|i\rangle \langle i|+\sum_{ij,j\neq i}t_{ij}|i\rangle \langle j|,$$

where $\epsilon_{i}$ is the on-site energy in QD${}_{i}$ and $t_{ij}$ is a factor that controls the hopping of an electron between QDs *i* and *j*.

Finally, using the second quantization approach, the Hamiltonian reads as

(44) $${\hat{H}}_{S,SQ}=\sum_{ij,j\neq i}t_{ij}|i\rangle \langle j|.$$

### 4.3. Generating the Network Associated to S System

With the aim of generating the network associated to the proposed quantum system, *S*, we have first to identify nodes and links. *S* consists of a number of elements (QDs) that are interconnected with each other (when the electron is allowed to hop between QDs).

Each QD in the system represents a node in the network. To simplify the notation, we encode any node *i* by using its corresponding electron state vector |i〉.

While discerning what a node is has been easy (QD ↔ node ↔ ket), more physical intuition is required to determine how the links are formed in such a way that they have physical meaning. A link can be formed if the electron is allowed to hop from one node to the other. If the involved nodes have the same energy, it is required that their wave functions overlap enough for the electron to tunnel between them. If the nodes have different energy, it is additionally necessary to include Boltzmann factors. It is convenient to keep in mind that, when considering the quantum system *S* in thermodynamical equilibrium at temperature *T* with the huge reservoir *E*, the energy interchange between them is the fact that allows electron hoppings between QDs *j* and *n* with small energy differences $\epsilon_{j}-\epsilon_{n}$. As explained in Section 3, the transitions rates $\gamma_{n\to j}$ and $\gamma_{j\to n}$ in a stationary state have to fulfill the detailed balance condition (22), which causes (1) Boltzmann factors to arise naturally, and (2) the incoherent part of the LME to vanish. This suggests that using coherent quantum dynamics makes physical sense. With this in mind, the possible hopping of an electron from QD *i* (with energy level $\epsilon_{i}$) to *j* (with energy level $\epsilon_{j}$), as shown in Figure 1h, is encoded by using a weighted link. We generate the link between two nodes (sites, kets), |i〉 and |j〉, located at $r_{i}$ and $r_{j}$ (with, in general, different energy levels, $\epsilon_{i}$ and $\epsilon_{j}$), by computing to what extent their respective wave functions overlap and by computing the corresponding Boltzmann factor so that the weight is

(45) $$w_{ij}=O_{ij}\cdot F_{B,ij},$$

where $O_{ij}$ can be computed in terms of the wave functions in the QDs using Equation (42), while $F_{B,ij}$ arises from Equation (22).

To advance in our model, it is necessary to introduce some essential network concepts. The first arises from the very interaction between nodes. When two nodes are *directly* connected by a link, they are said to be “adjacent” or neighboring. The adjacency matrix A encodes the topology of a network; that is, whether or not there is a link ($a_{ij}=1$ or $a_{ij}=0$) between any two pairs of nodes *i* and *j*. Sometimes, this binary information encoding whether or not a node is connected to another is not enough, and it is necessary to quantify the “importance” of any link (the strength of a tie between two users in a social network, or the flow of electricity between two nodes in a power grid [31]) by assigning a weight to each link. In that case, the matrix that encodes the connections is called a *weighted adjacency matrix* W [90].

The adjacency matrix corresponding to our system of *N* QDs is an N×N weighted adjacency matrix W whose matrix elements are

(46) $${\left(W\right)}_{ij}=\left\{\begin{array}{cc} 0 & ,\mathrm{if}\;i=j\\ O_{ij}\cdot F_{B,ij} & ,\mathrm{if}\;i\neq j \end{array}\right.$$

Once we have defined W in (46), we now have enough knowledge to represent the system as a network by using the undirected, weighted graph G≡G(N,L,W), where N is the set of nodes (card(N)=N) and L is the set of links. We have specified the matrix W in the triplet G≡G(N,L,W) to emphasize the fact that the connections between the nodes are made using the W matrix and not, for example, a conventional adjacency matrix A ($a_{ij}=1$ if *i* and *j* are directly linked; 0 otherwise), which would result in different results. Note that, because of the way we have generated the links, the weighted adjacency matrix W*quantifies* connections that have physical meaning according to quantum and statistical physics and explicitly includes the spatial structure of the system.

W helps us to obtain Laplacian matrices that will assist us in studying electron dynamics using CTQW, quantum walks that are continuous in time and discrete in space; see [55] for a very illustrative discussion on CTQW and their use in the simulation of quantum systems. There are several classes of Laplacian matrices [39,91].

The first type of Laplacian matrix, the (combinatorial) Laplacian—or simply Laplacian matrix—is defined as

(47) L=D−W,

where D is the diagonal degree matrix, whose elements $D_{i}$ are the sum of weights of all links directly connecting node *i* with others: $D_{i}=\sum_{i\neq j}{\left(W\right)}_{ij}$.

Note that the Laplacian matrix L computed using the weighted adjacency matrix W is different from the one used in other works [54,67,92]. In these approaches, the matrix elements of L are assumed to be equal $\gamma_{ij}\equiv \gamma =1$. In our approach, the matrix elements take different values as they depend on the involved overlap integrals and Boltzmann factors ($0\leq w_{ij}<1$) and, as shown throughout the paper, they play a natural role in the probability for an electron to hop from one node to another. The Laplacian acts as a node-to-node transition matrix so that the Hamiltonian of the CTQW can be written as H=L [50,58,60,65,67,68,93,94,95,96,97,98].

In particular, in our proposal, $L=H_{S,SQ}$, the matrix form of the TB Hamiltonian in the second quantization—Equation (44)—with $t_{ij}=w_{ij}$. Thus, the the unitary time evolution operator in quantum mechanics

(48) $$\hat{U}\left(t\right)=e^{-iH_{S,SQ}t/\hbar }$$

is equivalent to

(49) $$\hat{U}\left(t\right)=e^{-iLt/\hbar }.$$

The second very useful Laplacian is the normalized Laplacian matrix [93], $L_{N}=$$D^{-1/2}LD^{-1/2}$, a Hermitian operator that allows the generation of the corresponding unitary CTQW [93] of an electron on our graph G≡G(N,L,W) as

(50) $${\hat{U}}_{L_{N}}\left(t\right)=e^{-iL_{N}\cdot t}$$

Note that, in the time evolution operator generated by $L_{N}$ in (50), the imaginary unit makes ${\hat{U}}_{L_{N}}$ unitary [58]. As in other CN approaches [57,59,99,100], we assume ℏ≡1, meaning that time and energy can be treated as *dimensionless*. We use ${\hat{U}}_{L_{N}}\left(t\right)$ to study the temporal evolution of our quantum system.

## 5. Simulation Work

### 5.1. Methodology

As mentioned in the introduction, the *S* system contains two types of disorder. The first of them is determined by the fact that the QDs are located in a random way by means of the algorithm described in Section 4.2 to fulfill the minimum inter-dot distance condition. The second type of disorder has its origin in the fact that, in real QD layers, there are thermodynamic fluctuations in the dot size, leading to nodes with different energy levels. We have assumed the energy level distribution to be a Gaussian distribution with mean $\mu =E_{QD}=0.4$ eV and standard deviation $\sigma =10^{-3}$ eV. Thus, each QD (node) *i* is described by its corresponding $\epsilon_{i}$ energy level, a sample from the N(μ,σ). This energy level $\epsilon_{i}$ acts as an attribute called the hidden variable or fitness (see [47] for details in the context of SN).

With the aim of obtaining statistical values, we generated ensembles of networks with a sufficiently large number of networks. In the experiments, this led us to the conclusion that it was sufficient to generate 50 realizations of each complex network.

### 5.2. Testing the Weak Overlap Hypothesis

**Figure 3:**
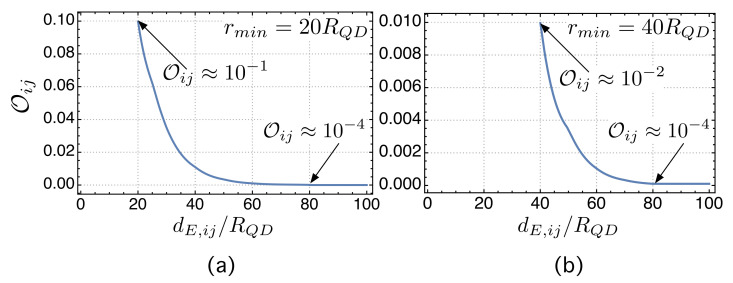
(**a**) Mean value of the overlap as a function of the normalized inter-dot distance $d_{E,ij}/R_{QD}$ in the case in which $r_{min}=20\;R_{QD}$. (**b**) Mean value of the overlap as a function of $d_{E,ij}/R_{QD}$ in the case in which $r_{min}=40\;R_{QD}$.

We begin this experimental section by aiming to verify whether the small overlapping hypothesis (stated by (42)) is true or false. In this respect, Figure 3 shows the mean value of the overlaps as a function of the normalized distance between dot centers ($d_{E,ij}/R_{QD}$) in two cases. The first one, in Figure 3a, corresponds to networks in which $r_{min}=20\;R_{QD}$. All possible overlaps are $O_{ij}<10^{-1}$, at least 1 order of magnitude lower than 〈i|i〉=1. Figure 3b represents the study case in which $r_{min}=40\;R_{QD}$ and shows how all the overlaps are $O_{ij}<10^{-2}$, at least 2 orders of magnitude below 〈i|i〉=1. These experimental results confirm that the overlapping integrals are small enough for the proposed model to be valid. We have also marked in Figure 3 the normalized distance $d_{E,ij}/R_{QD}=80$ for which the overlap is $O_{ij}\leq 10^{-4}$. In our model, we consider that no link is formed for longer distances.

### 5.3. Influence of the Minimum Inter-Dot Distance on Quantum Transport

As mentioned in the introduction, the motivation for this work is to understand what happens in a disordered QD system that is constrained to spatial (there is a minimum distance between dot centers to avoid localization in clusters) and physical restrictions (related to overlap integrals and Boltzmann factors). A first approach to this goal is to explore how an electron evolves in the generated network with time. Regarding this, we can characterize the network’s transport efficiency by using the average return probability (ARP), $\bar{\alpha }\left(t\right)$, defined as [68]

(51) $$\bar{\alpha }\left(t\right)=\frac{1}{N}\sum_{j=1}^{N}{\left|\langle j|{\hat{U}}_{L_{N}}\left(t\right)|j\rangle \right|}^{2},$$

where the operator ${\hat{U}}_{L_{N}}\left(t\right)$, presented in Equation (50), is the unitary time evolution operator governing the evolution of the probability amplitudes. Please note that, as shown in a number of papers [50,58,60,65,67,68,93,94,95,96,97,98], the Hamiltonian of the network is the Laplacian matrix (also called the connectivity matrix in some contexts). We have also shown that $L=H_{S,SQ}$, the matrix form of the TB Hamiltonian in the second quantization—Equation (44)—with $t_{ij}=w_{ij}$, meaning that the unitary time evolution operator in quantum mechanics is equivalent to ${\hat{U}}_{L_{N}}\left(t\right).$

High values of $\bar{\alpha }\left(t\right)$ suggest inefficient transport since the quantum particle tends to remain at the initial node [68]. On the contrary, $\bar{\alpha }\left(t\right)\ll 1$ means that the electron localized at the initial node in t=0 tends to be delocalized, with different (although similar) probability components on each node.

With this concepts in mind, we define the quantum transport efficiency (QTE) as [46]

(52) $$\eta_{QT}\left(t\right)=1-\bar{\alpha }\left(t\right).$$

**Figure 4:**
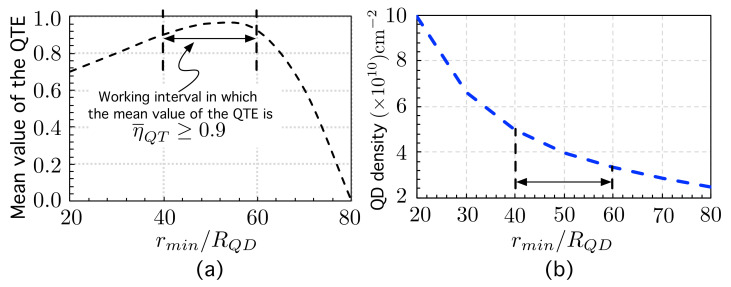
(**a**) Mean value (over 50 networks) of the quantum transport efficiency (QTE) as a function of the minimum inter-dot distance normalized by $R_{QD}$, $r_{min}/R_{QD}$. (**b**) QD density $(\times 10^{10}$ cm${}^{-2})$ as a function of $r_{min}/R_{QD}$.

Figure 4a shows the mean value (computed over 50 networks) of the quantum transport efficiency $\eta_{QT}$ stated by (52) as a function of the minimum inter-dot or exclusion radius $r_{min}$ normalized by $R_{QD}$.

Figure 4a provides interesting information: there is a value of the minimum normalized inter-dot distance ($r_{min}/R_{QD}$) for which the efficiency reaches a maximum value: ${\bar{\eta }}_{QT}=0.95$ for $r_{min}/R_{QD}=50$. For practical purposes, we have marked a “working interval” in which the mean value of the QTE is ${\bar{\eta }}_{QT}\geq 0.90$. The global behavior of ${\bar{\eta }}_{QT}$ can be explained as follows. For small values of $r_{min}/R_{QD}$, ranging from 20 to 40, increasing the minimum allowed inter-dot distance leads to a progressive reduction of QD clusters. The smaller the number of clusters, the higher the QTE. On the contrary, for high values of the minimum inter-dot distance (>$60\;R_{QD}$), the overlaps begin to be so small that the electron tends to be localized again. In particular, when approaching the maximum distance $r_{MAX}\equiv 80R_{QD}$—at which the proposed method no longer allows the formation of links ($O_{ij}\leq 10^{-4}$)—then ${\bar{\eta }}_{QT}\to 0$. This is because the QDs are so far apart that there is no interaction between them, or equivalently, the network nodes are disconnected.

These results can be expressed in terms of the QD density, as shown in Figure 4b. The interval of normalized inter-dot distances $r_{min}/R_{QD}\in \left(40,60\right)$ for which ${\bar{\eta }}_{QT}\geq 0.90$ leads to a QD density that ranges between $3.3\times 10^{10}$ cm${}^{-2}$ and $4.9\times 10^{10}$ cm${}^{-2}$, which would require a very precise control of the QD density.

**Figure 5:**
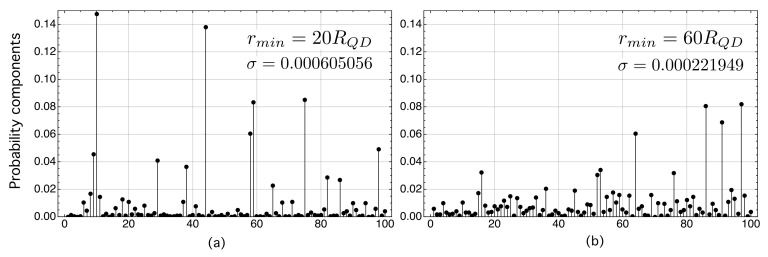
(**a**) Electron probability components, ${\left|\langle n|\psi \rangle \right|}^{2}$, on each of the kets |n〉 of a connected network with N=100 nodes. (**a**) Probability components for networks with $r_{min}=20R_{QD}$. (**b**) Probability components for networks with $r_{min}=60R_{QD}$.

Figure 5 is an example of the electron probability components, ${\left|\langle n|\psi \rangle \right|}^{2}$, on each of the kets |n〉 of a network with N=100 nodes. In this example, the electron was localized in node |10〉 at t=0 (initial state), and after applying the unitary evolution operator (50) for t=500, it evolved to an extended wave function whose probability components ${\left|\langle n|\psi \rangle \right|}^{2}$ were distributed over the N=100 nodes. We have considered two study cases that differ in the value of $r_{min}$. The first one corresponds to $r_{min}=20R_{QD}$ (Figure 5a), while the second one corresponds to $r_{min}=60R_{QD}$ (Figure 5b). These figures corroborate the previous results shown in Figure 4a. On the one hand, Figure 5a corresponds to a situation in which the excessive proximity between QDs ($r_{min}=20R_{QD}$) makes some of the dots localize the electron wave function more than others. On the other hand, in Figure 5b, corresponding to $r_{min}=60R_{QD}$, for which ${\bar{\eta }}_{QT}=0.90$, the probability components are better distributed than in study case (a), although not perfectly (as this would correspond to a crystalline solid). Note in this respect that the variance of the study case (b) is approximately 2.7 times smaller than that of case (a). This means that the electron wave function in the system with $r_{min}=60R_{QD}$ is more evenly distributed among the sites. This may have beneficial properties in photon absorption, requiring that the overlaps between the wave functions of the initial and final states be large. We present this discussion later in Section 6 as it requires some preliminary reasoning.

**Figure 6:**
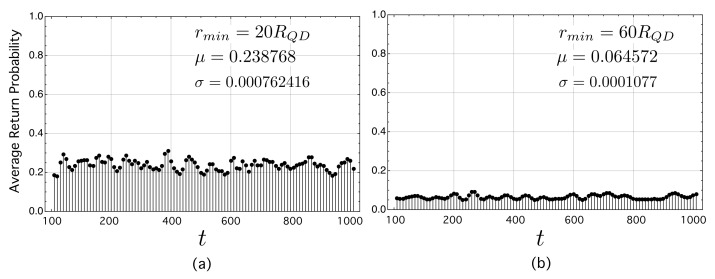
Average return probability (ARP) as a function of time (adimensional). As we have assumed ℏ≡1, then time and energy can be treated as dimensionless. Each value has been obtained as the mean value of the ARP over 50 networks with N=100 nodes each. (**a**) ARP for an ensemble of networks with $r_{min}=20R_{QD}$. (**b**) ARP for an ensemble of networks with $r_{min}=60\;R_{QD}$.

Figure 6 represents the ARP as a function of time (ARP-*t*) with t∈(100,1000) in two study cases. In both cases, the methodology is as follows. First, a network is generated and the ARP is computed for each time t∈(100,1000). Second, the first step is repeated for 50 instances of networks. Third, the final result is the average of the ARP-t over the 50 networks. The first study case in Figure 6a corresponds to 50 networks with $r_{min}=20R_{QD}$, while the second one corresponds to 50 networks with $r_{min}=60\;R_{QD}$ (Figure 6b). The analysis of these figures confirms again the beneficial properties of having minimum inter-dot distances in the “working” interval for which QTE ${\bar{\eta }}_{QT}\geq 0.90$. Specifically, the variance of ARP–*t* for $r_{min}=60R_{QD}$ is 7 times less than that corresponding to the case with $r_{min}=20R_{QD}$. In addition, the mean value of the ARP in (b), the ensemble of 50 networks with $r_{min}=60R_{QD}$, is very small, σ=0.0001077. This means again that the probability for an electron to hop through the network be high, preventing it from being localized in QD.

## 6. A Prospective Application

The modeling of the light absorption in semiconductors can be found in a number of works [101,102,103]. In this work, we have followed the most general point of view explained in [101]. It models the light absorption from the perspective of considering the system to be divided into two sub-systems: first, the electron system that consists of a set of quantum states and its corresponding energies; second, the photon system. We have also adopted this formulation and applied it to our QD system.

The absorption and emission of photons that cause electron transitions are essentially scattering events between initial electron states |i〉 and final states |f〉. The electromagnetic radiation is the perturbation that induces these events. We refer to the Hamilton operator that describes the electron–photon interaction as $H_{e-pt}$. Following Fermi’s Golden Rule, the transition rate from an initial electron state |i〉 to a final state |f〉, $\varrho_{i\to f}$, is given by the expression

(53) $$\varrho_{i\to f}\equiv \frac{1}{\tau }=\frac{2\pi }{\hbar }\sum_{f}{\left|\langle f|{\hat{H}}_{e-pt}|i\rangle \right|}^{2}\;\delta \left(E_{f}-E_{i}\pm \hbar \omega \right)$$

where τ is the lifetime that characterizes the transition, and the δ−function explicitly contains the photon energy ℏω. The upper sign of the ± labels the emission of this photon, and the lower sign shows its absorption.

As mentioned above, under the envelope approximation, any wave function Ψ can be expressed as $\Psi =\psi \;u_{B}$, where ψ is the envelope function and $u_{B}$ is the periodic part of the corresponding Bloch function for a *B* band. Therefore, the matrix element in Equation (53) becomes

(54) $$\langle f|{\hat{H}}_{e-pt}|i\rangle =\langle u_{f}|{\hat{H}}_{e-pt}{|u_{i}\rangle }_{cell}\langle \psi_{f}|\psi_{i}\rangle +{\langle u_{f}|u_{i}\rangle }_{cell}\langle \psi_{f}|{\hat{H}}_{e-pt}|\psi_{i}\rangle .$$

In inter-band transitions (see Figure 1e,f) between states in the conduction and valence bands (or between states derived from these bands by means of confinement potentials in low-dimensional structures), the second term on the right-hand side of the matrix element in (54) is zero. This is because the periodic part of the Bloch functions in two different bands, $u_{C(V)}$, at the same point in the Brillouin zone, are orthogonal: $\langle u_{f}|u_{i}\rangle \equiv \langle u_{C}|u_{V}\rangle =0$. Note that this means that the overlap integral $\langle \psi_{f}|\psi_{i}\rangle$ determines which transitions are allowed and which are forbidden.

On the contrary, in inter-sub-band or intra-band transitions, the first term on the right-hand side of the matrix element in Expression (54) is zero. The physical reason for this is that the envelope functions $\psi_{f}$ and $\psi_{i}$ are both eigenfunctions of the same Hermitian operator (the Conduction Band Hamiltonian), with different eigenvalues, and, as a consequence, $\psi_{f}$ and $\psi_{i}$ are orthogonal: $\langle \psi_{f}|\psi_{i}\rangle =0$.

Thus, the matrix elements of $H_{e-pt}$ can be written more compactly as

(55) $$\langle f|{\hat{H}}_{e-pt}|i\rangle =\left\{\begin{array}{cc} \langle u_{f}|{\hat{H}}_{e-pt}{|u_{i}\rangle }_{cell}\langle \psi_{f}|\psi_{i}\rangle , & \mathrm{inter}-\mathrm{band}\\ {\langle u_{f}|u_{i}\rangle }_{cell}\langle \psi_{f}|{\hat{H}}_{e-pt}|\psi_{i}\rangle , & \mathrm{intra}-\mathrm{band} \end{array}\right.$$

In the case of an isolated QD in Figure 1f, we can expect the intra-band transition from the CB discrete level (the electron intermediate level $E_{e,1}$ within the dot) up to a high energy state in the CB continuum to be weak because it would take place between a localized (discrete) state and a delocalized (continuum) state. In contrast, the photon absorption via an inter-band transition between a discrete energy level ($E_{h,1}$) in the valence band confinement potential (VB-CP) and a discrete energy level ($E_{e,1}$) in the CB confinement potential (CB-CP) can be significant because, aside from selection rules, the overlap occurs in the same region of space: the QD region. This is why the recombination between a confined electron ($E_{e,1}$) and a confined hole ($E_{h,1}$) within a QD can be radiative in nature, thus making the existence of QD lasers possible [13,14].

Different works using non-periodic QD distributions have been able to demonstrate the principles of operation of the QD-IBSC [22,23,24,25] but with the problem of a weak intra-band absorption ($E_{e,1}\to E_{e.2}$). With the aim that the intermediate states could be transformed into a band in which the wave function is completely delocalized, it has always been considered from a theoretical point of view that a sufficiently dense array of QDs would allow electrons to be coupled and delocalized enough to have strong absorption, causing transitions from the intermediate states to the CB states.

However, in the present work, we have shown that a very high density may not always be the best solution. Our work suggests that there is an optimal inter-dot distance (and consequently, an optimal dot density) for which the QTE is maximized, with the electron probability components at the dots becoming more uniform. However, in a very embryonic stage, we can have an intuition as to why this happens if we remember that the matrix elements of the electron–photon Hamiltonian [101,102] fulfill

(56) $$\langle f|{\hat{H}}_{e-pt}{|i\rangle }_{intra}\propto \langle \psi_{f}|\hat{e}\cdot ▽|\psi_{i}\rangle .$$

where $\hat{e}\cdot ▽$ is the gradient caused by the photon polarization vector $\hat{e}$. In the case of frontal sunlight illumination, $\hat{e}$ has only components in x and y. If the wave function in the initial intermediate state $|\psi_{i}\rangle$ is very confined in a QD (or in a few QDs), then the gradient $\hat{e}\cdot ▽|\psi_{i}\rangle$ varies strongly in that zone and very little in the rest. Consequently, the overlap of $\hat{e}\cdot ▽|\psi_{i}\rangle$ with the final wave function $|\psi_{f}\rangle$ (which is extended) is expected to be very small. Note that a final state within the CB is an extended function that is more similar to $u_{C}$ (the periodic part of the Bloch function in the CB) than $\hat{e}\cdot ▽|\psi_{i}\rangle$.

**Figure 7:**
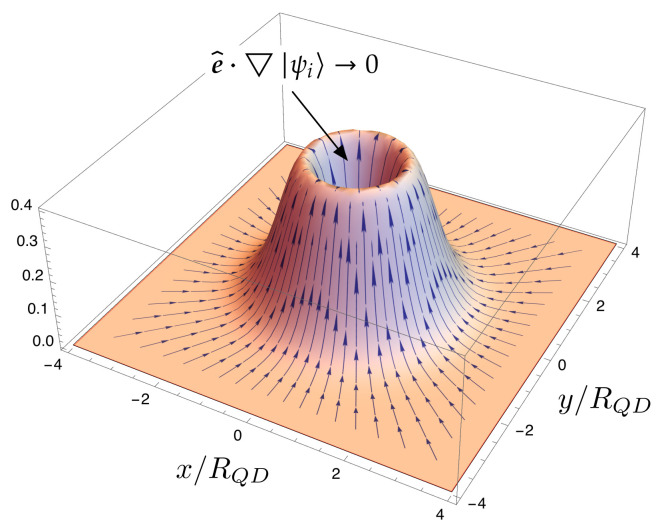
Gradient of electron wave function, $\hat{e}\cdot ▽|\psi_{i}\rangle$, in the dot. Note that $\hat{e}\cdot ▽|\psi_{i}\rangle \to 0$ when approaching the dot center.

**Figure 8:**
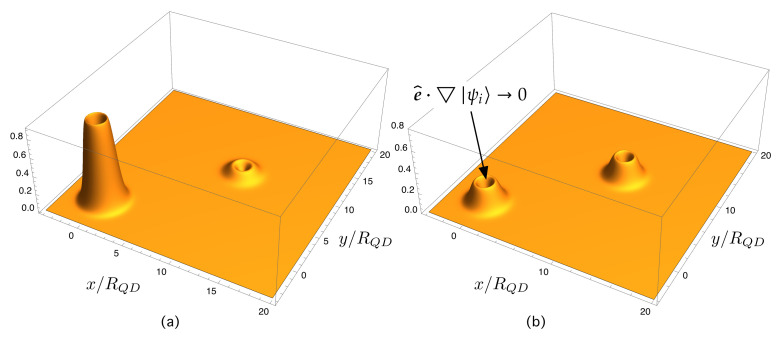
Gradient of electron wave function, $\hat{e}\cdot ▽|\psi_{i}\rangle$, in two cases. (**a**) Gradient of an initial state in which the probability components are very unbalanced. Its overlap with an extended final function on the continuum is expected to be very small. (**b**) Gradient of an initial state in which the probability components are unevenly distributed. Its overlap with an extended final function on the continuum is expected to be greater than in the case (**a**).

For illustrative purposes, Figure 7 and Figure 8 assist in explaining these ideas intuitively. Figure 7 shows the gradient of the electron wave function, $\hat{e}\cdot ▽|\psi_{i}\rangle$, in a dot located at (0,0). Note that $\hat{e}\cdot ▽|\psi_{i}\rangle \to 0$ when approaching the dot center. This is expected to produce a small overlap with the final extended function in the CB continuum. Figure 8 shows the gradient in two illustrative cases. The first one, represented in Figure 8a, shows the gradient of an initial state in which the probability components are very unbalanced. The overlap with an extended final function on the continuum is expected to be very small. Figure 8b illustrates the gradient of an initial state in which the probability components are unevenly distributed. The overlap with an extended final function on the continuum is expected to be higher than in the previous case.

Thus, if the wave function in the initial state is better balanced throughout the QD layer (as shown in Figure 5)—avoiding the existence of clusters with dots that are too close, which tend to confine the electron—then its gradient is expected to be smoother and better distributed and, consequently, a higher overlap with the fully extended final wave function is expected. Regarding this conclusion, which needs to be reinforced in more complex future work, special care should be taken in growth processes to ensure that there are no areas in which there is a high density of QDs to the detriment of others where the density is lower.

## 7. Summary and Conclusions

This paper has proposed the modeling of a quantum system, *S*, made up of *N* disordered quantum dots (QDs), by using complex networks (CN) with spatial and physical-based constraints. The disorder is twofold: on the one hand, the QDs are randomly distributed; on the other hand, the sizes of the QDs may vary slightly. While discerning what a node is seems easy (QD ≡ node), more care and physical intuition is required when determining how the links between QDs are formed in such a way that they have physical meaning. In this respect, the novelty of our model is threefold: first, although we have considered the QDs (=nodes) to be randomly distributed in a metric space, they have to fulfill the key condition that there is a minimum distance between dot centers ($r_{min}$) that cannot be violated (to prevent the electron from being localized in some QDs in detriment of others); second, our model allows nodes with different attributes to be considered—in particular, with different energy levels; third, the link formation and the weighting process that we have proposed are consistent with the laws of quantum physics and statistics.

Put simply, given two QDs *i* and *j* with the same energy level, the probability of an electron tunneling between them is related to the corresponding overlap integrals. If, additionally, the dots have slightly different energy levels, the probability of the electron hopping between them is related to Boltzmann factors. We have tested the consistency of our approach in the theoretical framework stated by the Lindblad master equation (LME). The LME allows the study of the quantum dynamics of the reduced density matrix operator of our open quantum system *S*, which is in thermodynamic equilibrium with a much bigger environment or reservoir *E* at a given temperature *T*. The electron stationary state is reached under detailed balance conditions that make the LME incoherent part vanish, from which the Boltzmann factors naturally arise: when an electron hops from a QD with energy $\epsilon_{n}$ to another with energy $\epsilon_{j}>\epsilon_{n}$, the energy difference $\Delta E=\epsilon_{n}-\epsilon_{j}$ is supplied by the environment. The opposite is also found to be true using the mentioned detailed balance concepts in the canonical ensemble. The fact that the incoherent part vanishes allows us to consider coherent quantum dynamics.

Our method to generate links leads to a weighted adjacency matrix whose elements contain overlap integrals and Boltzmann factors. The corresponding Laplacian matrix L, which assists in computing continuous time quantum walks (CTQW) on the associated network, is different from the model used in other works [54,67,92]. In these approaches, the matrix elements of L are assumed to be equal $\gamma_{ij}\equiv \gamma =1$. In our approach, the matrix elements take different values since they depend on the involved overlap integrals and Boltzmann factors, and, as shown throughout this paper, they play a natural role in the probability for an electron to hop from one node to another.

Specifically, in our approach, the matrix form of the tight-binding (TB) Hamiltonian in the second quantization is $H_{S,\;SQ}=L$, meaning that the corresponding time evolution unitary operators are equivalent.

The simulation work we have carried out focused on two key points. Firstly, we tested the weak overlap hypothesis that is necessary for the TB to be used. We studied the mean value of the overlap integrals for different values of $r_{min}$, the minimum distance between centers. Networks with $r_{min}\in \left(40R_{QD},\;60R_{QD}\right)$ have overlap integrals $O_{ij}<10^{-2}$ that are at least 2 orders of magnitude below 〈i|i〉=1. These experimental results confirm that the overlapping integrals are small enough for the proposed model to be valid.

The second group of simulation work aimed to explore the influence of the minimum inter-dot distance $r_{min}$ on the quantum transport efficiency (QTE) and on the electron probability distributions. The main result was that a value was found for the minimum normalized inter-dot distance ($r_{min}/R_{QD}\approx 50$) for which the mean value (over 50 networks) of the QTE is maximum: ${\bar{\eta }}_{QT}=0.95$. There is also a “working interval” for $r_{min}\in \left(40R_{QD},60R_{QD}\right)$ for which ${\bar{\eta }}_{QT}\geq 0.90$. In this interval, the electron probability components have been found to be more smoothly distributed than in those cases in which QDs are allowed to be very close. We have explained the global behavior of ${\bar{\eta }}_{QT}$ as follows. For small values of $r_{min}/R_{QD}$, ranging from 20 to 40, increasing the minimum allowed inter-dot distance leads to a progressive reduction of QD clusters. The smaller the number of clusters, the higher the QTE. On the contrary, for high values of the minimum inter-dot distance (>$60\;R_{QD}$), the overlaps begin to be so small that the electron tends to be localized again. In particular, when approaching the maximum distance $r_{MAX}\equiv 80R_{QD}$—for which the proposed method no longer allows the formation of links ($O_{ij}\leq 10^{-4}$)—then ${\bar{\eta }}_{QT}\to 0$. This is because the QDs are so far apart that there is no interaction among them, or equivalently, the network nodes are disconnected. These results can be expressed in terms of the QD density. The interval of $r_{min}/R_{QD}\in \left(40,60\right)$, for which ${\bar{\eta }}_{QT}\geq 0.90$, leads to a QD density ranging between $3.3\times 10^{10}$ cm${}^{-2}$ and $4.9\times 10^{10}$ cm${}^{-2}$, which would require a very precise control of the QD density.

The existence of a value interval for $r_{min}$ in which the QTE is high and the electron wave function is distributed in a smoother way (although not perfectly) over the QDs could have consequences on light absorption processes. In particular, the model could explain why the photon absorption causing transitions from levels at the QDs to the conduction band (CB) has been found to be weak in quantum dot intermediate-band solar cells (QD-IBSCs). This is because the matrix elements of the electron–photon Hamiltonian ruling these transitions is proportional to $\langle \psi_{f}|\hat{e}\cdot ▽|\psi_{i}\rangle$, where $\psi_{i}$ and $\psi_{f}$ are, respectively, the envelope wave functions for the electron in the initial state (in the bound state in the dot) and the final state (in the CB continuum), and $\hat{e}\cdot ▽$ is the gradient operator caused by the photon polarization vector $\hat{e}$. In the case of frontal sunlight illumination, $\hat{e}$ has only components in *x* and *y*. If the wave function in the initial intermediate state $|\psi_{i}\rangle$ is very confined in a QD (or in a few QDs), then the gradient $\hat{e}\cdot ▽|\psi_{i}\rangle$ varies strongly in that zone and very little in the other areas. Consequently, the overlap of $\hat{e}\cdot ▽|\psi_{i}\rangle$ with the final wave function $|\psi_{f}\rangle$ (which is extended) is expected to be very small. We think that if the wave function in the initial state is better balanced throughout the QD layer—avoiding the existence of QD clusters that are prone to confine the electron—then its gradient in *x* and *y* is expected to be smoother and better distributed. As a consequence, a higher overlap with the fully extended final wave function in the CB is expected. Regarding this thought experiment, which needs to be proved in future work, special care should be taken in growth processes to avoid the formation of clusters.

## Data Availability

Not applicable.

## Abbreviations and Nomenclature

### Abbreviations

The following abbreviations are used in this manuscript:

0D	Zero-dimensional
1D	One-dimensional
2D	Two-dimensional
ARP	Average Return Probability
CB	Conduction Band
CN	Complex Networks
CP	Confinement Potential
CTQW	Continuous-Time Quantum Walks
DM	Dot Material
GS	Ground State
IB	Intermediate Band
IBSC	Intermediate-Band Solar Cell
LCAO	Linear Combination of Atomic Integrals
LME	Lindblad Master Equation
QD	Quantum Dot
QD-IBSC	Quantum Dot Intermediate-Band Solar Cell
QM	Quantum Mechanics
QT	Quantum Transport
QW	Quantum Walk
RGG	Random Geometric Graph
RN	Random Network
SAQDs	Self-Assembled Quantum Dots
SML-QDs	Sub-Monolayer Quantum Dots
SN	Spatial Network
SK	Stranski–Krastanow
SW	Small World
TB	Tight-binding
VB	Valence band
VW	Volmer–Weber
WL	Wetting layer

### Nomenclature

𝟙	Identity operator in Quantum Mechanics.
A	Adjacency matrix of a graph G.
$a_{ij}$	Element of the adjacency matrix A.
$\bar{\alpha }\left(t\right)$	Average return probability.
D	Node degree matrix: $\mathrm{diag}(k_{1},\cdots ,k_{N})$. It is the diagonal matrix formed from the nodes degrees.
$d_{E}\left(i,j\right)$	Euclidean distance between any pair of nodes *i* and *j* in a network.
$d_{ij}$	Distance between two nodes *i* and *j*. It is the length of the shortest path (geodesic path) between them, that is, the minimum number of links when going from one node to the other.
$d_{E,Lim}$	$d_{E,Lim}\equiv d_{S}$ Euclidean distance limit beyond which there is no link formation.
$E_{e}$	Energy level of a confined electron in a quantum dot.
$E_{h}$	Energy level of a confined hole in a quantum dot.
$E_{QD}$	Discrete electron energy in a quantum dot (QD).
$\eta_{QT}$	Quantum transport efficiency.
G	Graph G≡G(N,L,W), where N is the set of nodes (card(N)=N), L is the set of links, and W is weighted adjacency matrix that emerges from our method to link formation.
$F_{B,ij}$	Boltzmann factor.
$\hat{H}$	Hamiltonian operator corresponding to the total energy of a quantum system.
H	Hamiltonian in matrix form.
*ℏ*	Reduced Planck constant.
H	Hilbert space.
$H_{eq}$	Equilibrium Hilbert subspace.
|i〉	Ket vector in the Hilbert space H.
〈i|	Bra vector in the *dual space* corresponding to the ket |i〉 ∈H
*ℓ*	Average path length of a network. It is the mean value of distances between any pair of nodes in the network.
L	Set of links (edges) of a network (graph).
L	Laplacian matrix of a graph G.
$L_{N}$	Normalizad Laplacian matrix, $L_{N}=$ $D^{-1/2}LD^{-1/2}$.
$m_{e}$	Electron mass.
*M*	Size of a graph G. It is the number of links in the set L.
*N*	Order of a graph G=(N,L). It is the number of nodes in set N, that is the cardinality of set N: $N=\left|N\right|\equiv \mathrm{card}\left(N\right)$.
N	Set of nodes (or vertices) of a graph.
${▿}^{2}$	Laplace operator.
P(k)	Probability density function giving the probability that a randomly selected node has *k* links.
|ψ〉	Ket or vector state in Dirac notation corresponding to the wave function ψ.
$R_{QD}$	Radius of the quantum dot.
$\psi_{QD}$	Electron wavefunction in a quantum dot.
$\tau_{B}$	Boltzmann time.
$\hat{V}$	Potential energy operator.
$-V_{C}$	Depth of confinement potential.
$U_{C}\left(r\right)$	Confining, spherical (depending only on the radial co-ordinate *r*), finite, and square potential energy.
${\hat{U}}_{L_{N}}\left(t\right)$	Time evolution operator generated by the normalizad Laplacian matrix $L_{N}$.
$w_{ij}$	Weight of the link between node *i* and *j*.
W	weighted adjacency matrix.
